# Acute myocarditis associated with COVID-19 vaccination: A case report

**DOI:** 10.1016/j.jccase.2021.11.006

**Published:** 2021-12-03

**Authors:** Takashi Nagasaka, Norimichi Koitabashi, Yohei Ishibashi, Kazufumi Aihara, Noriaki Takama, Yoshiaki Ohyama, Tomoyuki Yokoyama, Yoshiaki Kaneko

**Affiliations:** a Department of Cardiovascular Medicine, Gunma University Graduate School of Medicine, Maebashi, Japan; b Department of Laboratory Sciences, Gunma University Graduate School of Health Sciences, Maebashi, Gunma, Japan

**Keywords:** COVID-19, Vaccine, Myocarditis, Biopsy

## Abstract

Recently, new vaccine platforms-including mRNA vaccines for coronavirus disease 2019 (COVID-19) have been given emergency use authorization in Japan. Here, we present a rare case of myocarditis following a COVID-19 vaccine. In this case, myocarditis was confirmed by cardiac magnetic resonance imaging, endomyocardial biopsy, and troponin levels. The degree of myocardial inflammation in the endomyocardial biopsy samples was mild and the patient's clinical course was not severe. Although the pathology of myocarditis in this case was mild, further investigation would be needed.

<**Learning objective:** Vaccination for coronavirus disease 2019 is advancing worldwide, but post-vaccination myocarditis is getting attention as a rare side effect. Although the myocarditis in this case was mild, the pathogenesis of the disease is unclear and needs to be thoroughly investigated in the vaccination.>

## Introduction

Severe acute respiratory syndrome coronavirus 2 (SARS-CoV-2) is a virus that is the cause of a critical disease known as coronavirus disease 2019 (COVID-19). It is known that Covid-19 can cause severe respiratory infections. In addition, some cases of myocarditis in COVID-19 patients have been reported [1], and myocarditis has been recognized as the cause of death in some COVID-19 patients [2]. At the same time, there are limited reports on definite diagnosis of myocarditis caused by SARS-CoV-2 in humans and limited demonstration of the virus in the myocardium. Recently, new vaccine platforms for COVID-19 including mRNA vaccines and viral vector-based DNA vaccines have been given emergency use authorization, leading to mass vaccinations in Japan. The results of some trials revealed that the vaccine was effective, safe, and similar to that of other vaccines [3]. On the other hand, some trials showed adverse events after COVID-19 vaccine [3]. It is said that the most frequent adverse events were fever, fatigue, headache, myalgia, and injection-site pain [4]. However, few cases have reported vaccination as a possible trigger for myocarditis. We present a rare case of myocarditis following a COVID-19 vaccine.

## Case report

**Fig. 1:**
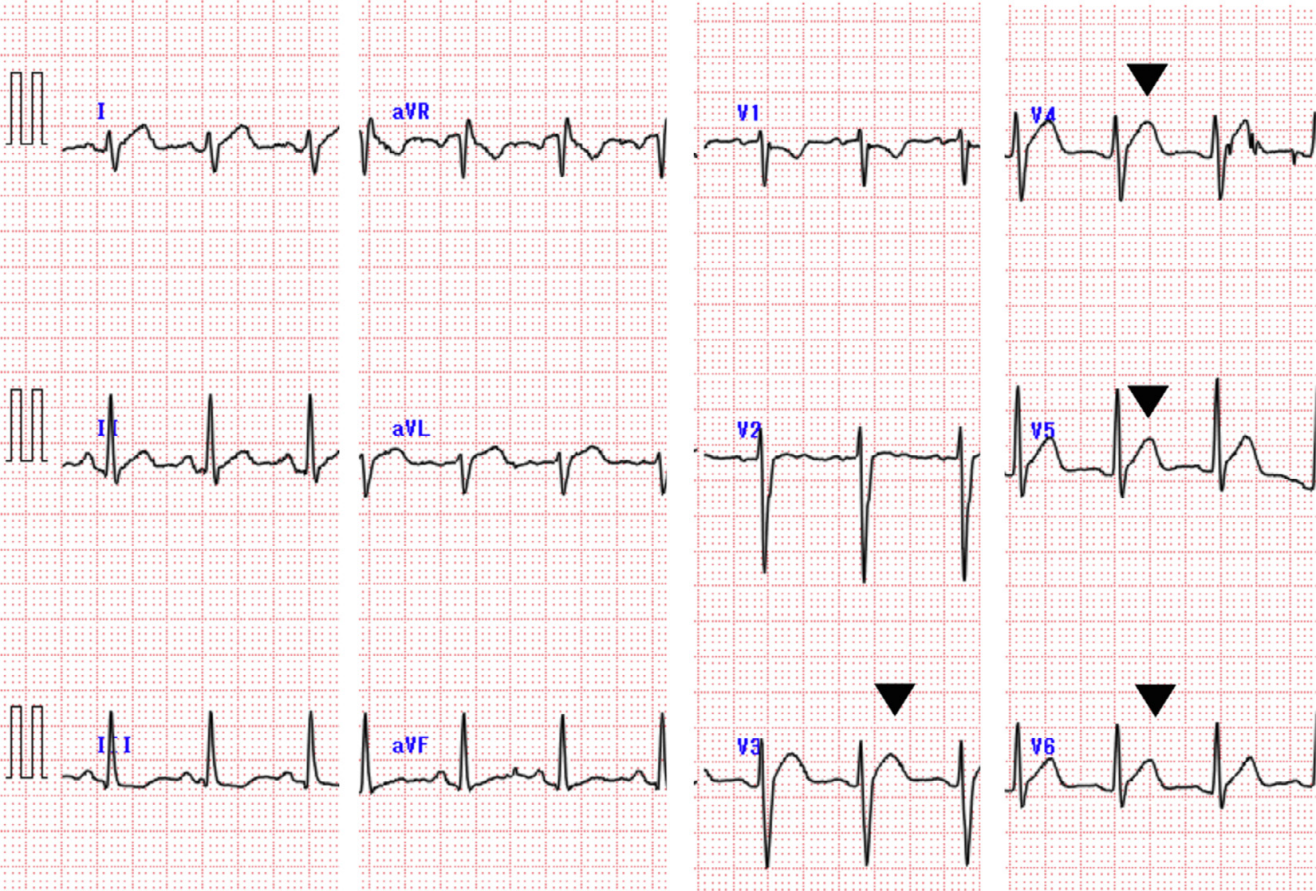
Electrocardiogram on admission. ST segment elevation was observed in the precordial leads (V3-V6, black arrow heads).

A 23-year-old man with no previous history of cardiovascular disease or any significant medical history presented to the emergency room with fever and chest pain without respiratory symptoms for 3 days after the second dose of a COVID-19 vaccine (Pfizer-BioNTech BNT16B2b2 mRNA vaccine). He was up-to-date on his vaccines, including yearly influenza, with no history of adverse reactions. He had no known COVID-19 exposure and his SARS-CoV-2 antigen and reverse transcriptase-polymerase chain reaction were negative on nasopharyngeal swab testing. On admission, his vital signs were: body temperature, 37.8°C; blood pressure, 106/70 mmHg; pulse, 83 beats/min; respiratory rate, 18 breaths/min; and oxygen saturation, 96% on room air. Initial blood samples showed increased troponin I levels: 4,550 pg/mL (normal value: ≤24 pg/mL) and C-reactive protein: 10.16 mg/dL (normal value: ≤0.1 mg/dL). His white blood cell count was 8,600/μL (normal value: ≤9,600/μL) without eosinophilia. Electrocardiography (ECG) revealed subtle ST elevation suggestive of potential myocardial injury or pericarditis in V3-V6 (Fig. 1). Transthoracic echocardiography showed segmental wall motion abnormality of the anteroseptal portion of the left ventricle with mild pericardial effusion. Chest radiography showed absence of pulmonary congestion, pleural effusion, and cardiomegaly. Emergency coronary angiography performed to rule out acute coronary syndrome demonstrated normal arteries. Hence, endomyocardial biopsy (EMB) was performed to diagnose the etiology.

**Fig. 2:**
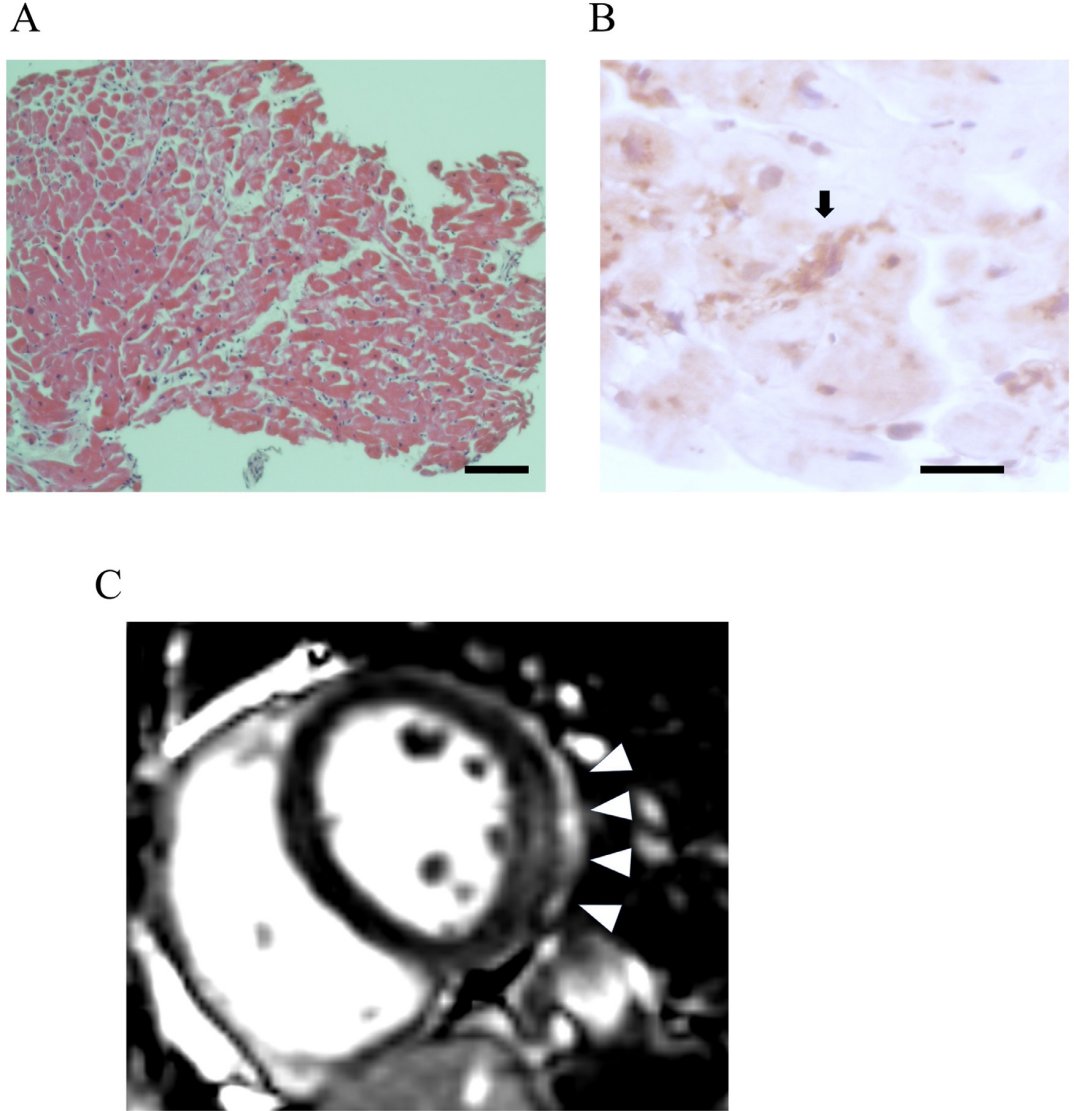
Acute myocarditis after COVID-19 vaccination. (A) Histopathological evaluation of an endomyocardial biopsy sample from the left ventricle. Hematoxylin-eosin staining showed a small amount of infiltration of mononuclear cells. Scale bar, 100 μm. (B) Immunostaining with CD68 antibody of mononuclear cells in the endomyocardial biopsy sample. Immunohistochemistry was performed with mouse monoclonal CD68 antibody (MCA341R, BIO-RAD laboratories, Hercules, CA, USA) using the ABC kit (Vector Laboratories, Burlingame, CA, USA) according to the manufacturer's protocol. CD68-positive cells are indicated with a black arrow. Scale bar, 25 μm. (C) Cardiac MRI imaging. Short-axis view of a post-contrast image showing sub-epicardial and mid-myocardial late gadolinium enhancement (white arrow heads) in the mid-chamber inferolateral wall.

Light microscopy of EMB samples revealed a small amount of inflammatory cell infiltration composed of mononuclear cells (Fig. 2A). Immunostaining showed that some of the infiltrating inflammatory cells were CD68-positive and considered to be macrophages (Fig. 2B). On the other hand, CD3 positive cells were rarely seen in the biopsied tissue (data not shown). Additionally, cardiac magnetic resonance imaging (MRI) showed late gadolinium enhancement, predominantly sub-epicardial and mid-myocardial enhancement, mainly in the inferolateral walls, typical of acute myocarditis (Fig. 2C). These findings are suggestive of myocardial edema and pericardial inflammation, and were compatible with acute myocarditis [5]. He was treated with a nonsteroidal anti-inflammatory drug after admission. On the second day of hospitalization, his troponin I and creatine kinase levels in blood (CK-MB) were elevated [troponin I, 8,699 pg/mL; CK-MB, 13.7 U/L (normal value: ≤10 U/L)]. On day 5, echocardiography showed normalization of wall motion, and the laboratory findings had almost normalized to: troponin I, 174 pg/mL; CK-MB, 2.2 U/L; and C-reactive protein, 0.61 mg/dL. His symptoms had also simultaneously abated, and his troponin levels and echocardiogram had returned to almost normal. He was eventually discharged on day 7 of his hospital stay.

We examined for neutralizing antibody titers to coxsackie B1-6, echo 5, 6,7,9,11 viruses, parvovirus B19, cytomegalovirus, human herpesvirus, and Epstein-Barr virus in acute phase and chronic phase after 4 weeks. However, there were no significant rises in titer over a four-week period. During the hospitalization, we measured total immunoglobulin levels to the receptor-binding domain of the SARS-CoV-2 spike protein with an anti–SARS-CoV-2 S enzyme immunoassay (Elecsys, Roche Diagnostics International Ltd, Basel, Switzerland). The titer of SARS-CoV-2 antibodies in this patient was 627.0 U/mL, which was a sufficient increase in titer.

## Discussion

Myocarditis is an inflammatory disease of the heart characterized by inflammatory infiltrates and myocardial injury without an ischemic cause. The clinical diagnosis in this patient was acute myocarditis after COVID-19 vaccination. In this case, myocarditis was confirmed by cardiac MRI, EMB, and troponin levels. Some previous studies have also reported adverse events after COVID-19 vaccination [3]. Recently, several case reports have shown vaccination as a possible trigger for myocarditis [6], [7], [8], [9], [10]. However, to the best of our knowledge, there are no case reports of EMB used to diagnose myocarditis after COVID-19 vaccination in Japan. In this case, the degree of myocardial inflammation in the EMB samples was mild and the patient's clinical course was not severe. It has been reported that the inflammatory infiltrate in COVID-19 vaccine-induced myocarditis predominantly composed of T-cells and macrophages, admixed with eosinophils, B cells, and plasma cells [9]. Another report from Korea showed macrophage-dominance [10]. The pathology in our case was predominantly macrophages, but this pathology cannot explain the mechanism because the myocardial biopsy sample was small and had few inflammatory cells. Further investigations are needed to better understand the potential association between COVID-19 vaccines and myocarditis.

This report of mild myocarditis after COVID-19 vaccination probably represents a temporary post-vaccination adverse reaction. Clinicians need to be aware of the potential for cardiopulmonary symptoms after recent vaccination.

In conclusion, this report of myocarditis after COVID-19 vaccination may be probably considered as an adverse reaction following immunization. Although the pathology of myocarditis in this case was mild, further investigation of this non-negligible side effect is needed.

## Declaration of Competing Interest

The authors declare that there is no conflict of interest.
